# Aging of Plasma-Activated Polyethylene and Hydrophobic Recovery of Polyethylene Polymers

**DOI:** 10.3390/polym15244668

**Published:** 2023-12-11

**Authors:** Miran Mozetič

**Affiliations:** Department of Surface Engineering, Jozef Stefan Institute, Jamova Cesta 39, 1000 Ljubljana, Slovenia; miran.mozetic@guest.arnes.si

**Keywords:** polyethylene, wettability, aging, hydrophobic recovery, gaseous plasma

## Abstract

Available literature on the aging of plasma-activated polyethylene due to hydrophobic recovery has been reviewed and critically assessed. A common method for the evaluation of hydrophobic recovery is the determination of the static water contact angle, while the surface free energy does not reveal significant correlations. Surface-sensitive methods for the characterization of chemical composition and structure have limited applicability in studying the aging phenomenon. Aging is driven by thermodynamics, so it is observed even upon storage in a vacuum, and hydrophobic recovery increases with increasing temperature. Storage of plasma-activated polyethylene in the air at ambient conditions follows almost logarithmic behavior during the period studied by most authors; i.e., up to one month. The influence of the storage medium is somehow controversial because some authors reported aging suppression by storing in polar liquids, but others reported the loss of hydrophilicity even after a brief immersion into distilled water. Methods for suppressing aging by hydrophobic recovery include plasma treatment at elevated temperature followed by brief treatment at room temperature and application of energetic ions and photons in the vacuum ultraviolet range. Storing at low temperatures is a trivial alternative, but not very practical. The aging of plasma-activated polyethylene suppresses the adhesion of many coatings, but the correlation between the surface free energy and the adhesion force has yet to be addressed adequately.

## 1. Introduction

Products made from polymers, polymer blends, and polymer composites are widely used because of their relatively low weight, adequate mechanical and chemical properties, and ability to make products of complex shapes. The as-synthesized products are wettable enough to assure functional properties, but aging is unavoidable over longer periods. The aging is often accelerated by exposure to a harsh environment, such as chemical agents, irradiation with photons of energy larger than the binding energy between atoms or molecular fragments in the polymer structures (typically ultraviolet (UV) radiation), and gaseous species of high oxidation potential. The aging is often triggered by surface reactions, and the structural modification proceeds by chain initiation, propagation, branching, termination, etc. Numerous techniques for the detection of polymer aging have been used, and a recent review of available techniques, as well as their limitations, has been published as [1]. The initial stages, however, are challenging to evaluate, and sophisticated methods should be used [2].

The degradation of the functional properties of polymers upon and after exposure to ultraviolet (UV) radiation has been studied by numerous authors [3,4,5,6]. The UV photons absorb in the surface layer of polymer materials by breaking chemical bonds. The penetration depth depends on the type of polymer and the photon energy. As a rule of thumb, the penetration depth decreases with increasing photon energy [7]. The structural modification caused by UV photon absorption is often associated with surface oxidation; i.e., the irradiation with energetic photons breaks bonds, and surface oxidation occurs even at ambient conditions [8]. An alternative effect is cross-linking. The cross-linking is a known effect and is often used for curing (i.e., initiating photochemical reactions that generate a crosslinked network) some types of polymers [9]. The oxidation of polymer surfaces also occurs without exposure to energetic photons—for example, by exposure to ozone [10,11,12,13].

The surface properties of polymer products are not always adequate, so they have to be modified. The polymer materials are often exposed to gaseous plasma in order to tailor the surface properties. The surface is hydrophobized by fluorination [14] and/or deposition of a very thin hydrophobic film [15]. Such a surface treatment ensures low wettability, which is desired in many cases. However, in many other cases, the opposite effect should be achieved; i.e., the hydrophilization of polymer products. The increased wettability due to a highly hydrophilic surface finish is needed prior to the deposition of various coatings, including printing, gluing, and metallization. The increased wettability is usually obtained by exposure of the polymer product to gaseous plasma, which causes surface oxidation. A natural choice is the application of oxygen plasma, but plasmas of other gases will also cause oxidation upon exposure of the plasma-treated polymers to ambient conditions. For example, plasma sustained in the sulfur-containing gases is useful for improving the hemocompatibility of polyethylene [16]. Plasma hydrophilization of polymer materials remains a hot topic of surface science, and numerous articles have been published. Some reviews include [17,18,19,20].

As mentioned earlier, the aging of polymers at room temperature is often triggered by irradiation with UV photons of exposure to oxygen of high oxidation potential, like ozone, superoxide, and atomic oxygen. Oxygen plasmas are sources of both energetic photons and oxygen species of high oxidation potential, so it is likely that the surface hydrophilization by exposure of polymer materials to oxygen plasma will not be permanent, so aging will occur spontaneously after the plasma treatment. Oxygen plasma is a powerful source of radiation in the vacuum ultraviolet (VUV) range [21,22]. It also contains molecular and atomic ions, neutral atoms in the ground and excited states, and molecules in excited states [23]. The kinetics of surface oxidation as a result of the synergy of all plasma species is still not well understood, but some useful theories have been published recently [23]. The theories are partially supported by recent experiments on the evolution of polymer surface functionalization upon treatment with oxygen plasma [24,25]. The wettability is usually determined by measuring the contact angle of a water droplet (WCA). Some authors also deposit droplets of other liquids with known surface tension and calculate the polymer’s surface free energy (SFE).

**Figure 1:**
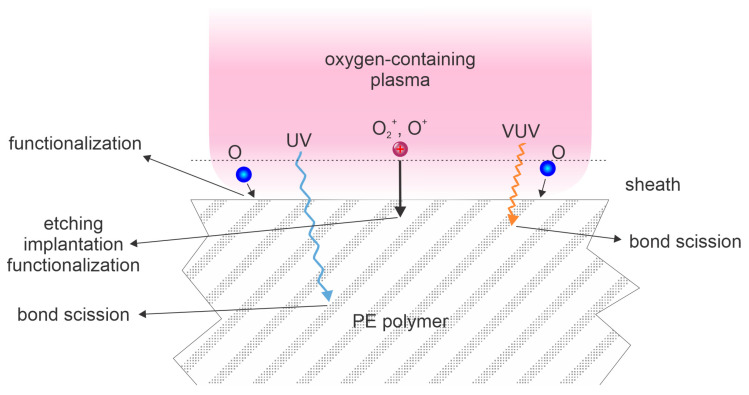
Illustration of polyethylene (PE polymer) activation by oxygen plasma. Oxygen plasma contains neutral oxygen atoms (O), which will interact chemically on the polymer surface only; ultraviolet (UV) radiation of rather large penetration depth; positively charged molecular (O_2_^+^) and atomic (O^+^) ions of very low penetration depth; and vacuum ultraviolet (VUV) radiation of moderate penetration depth. Positively charged ions are perpendicular to the surface and monochromatic in the collision-less sheath approximation, while the flux of other species is randomly distributed over surface collision angles.

Polyethylene is among the most widely used plastics because of its low cost, high ductility, low water permeability, and excellent chemical stability. Its wettability is poor because of the absence of polar surface functional groups, and thus, the polar component of the surface free energy is very low. The surface energy and the wettability are therefore increased by treatment with gaseous plasmas containing oxygen, including air. The treatment is illustrated in Figure 1. Plasma is rich in positively charged ions and neutral oxygen atoms, and highly excited molecules, radicals, and ions emit photons. There is a sheath between the plasma and polyethylene sample, and the positively charged ions accelerate in the electric field within the sheath so they bombard the PE surface. If the sheath voltage is large (i.e., when the sample is biased when placed onto an electrode powered by an AC voltage source), implantation of positive ions in the surface film occurs. If the sample is kept at the floating potential, the implantation depth is marginal, so both the potential and kinetic energies are predominantly spent on etching of the PE surface. The interaction with ions also causes surface functionalization; i.e., the substitution of C–H surface bonds with C–OH, C=O, O=C–OH, and other similar bonds. The oxygen molecules will partially dissociate in plasma conditions, and the O-atoms will be thermal, will diffuse in the treatment chamber, and may reach the polymer surface. Their kinetic energy is marginal since the neutral O atoms, unlike positively charged ions, do not feel the voltage across the sheath, so the predominant reaction is functionalization with polar functional groups. Plasma is also a source of radiation, and the penetration depth is low in the case of VUV, and moderate for UV photons [7]. The photons will cause bond scission if the photon energy is larger than the bond strength. Obviously, VUV photons will not be selective in breaking specific bonds, since their energy is larger than any bond energy in PE. The UV photons will predominantly absorb through scission of the weakest bonds. The bond scission may lead to surface degradation, formation of low molecular weight fragments, and lead to formation of debris on a polymer surface, especially after prolonged treatment [26]. Upon treatment with powerful inductively coupled plasma, the chain degradation in some polyolefins will also cause beta-scission [27].

The plasma treatment will cause increased surface wettability because of the formation of polar surface functional groups. The large concentration of the polar groups is not thermodynamically stable but aging (hydrophobic recovery) is usually observed. This paper reviews the scientific literature on the aging of both low and high-density polyethylene, and explains the observations reported by various authors.

## 2. Aging of Plasma-Treated Polyethylene

The hydrophobic recovery of polyethylene (PE) samples treated by gaseous plasma was probably first mentioned in 1993 in the classical work by Benisch et al. [28]. In this short article, the authors treated PE foils in a flowing afterglow of oxygen plasma. The type of the PE foils or the synthesis procedure was not mentioned. Experiments with gaseous plasma were performed in a Pyrex tube. Gas was introduced in the upper part of the tube and pumped to the lower part. Plasma was sustained in the upper part of the Pyrex tube using a capacitively coupled electrodeless RF discharge, powered with an RF generator operating at 13.56 MHz. The advancing and receding WCA were 106 and 87°, respectively. The WCA for a sample treated with an oxygen plasma afterglow for 30 s was about 40°, and increased to 70° after storing the sample for 10 min in a vacuum at 50 °C. Prolonged storage did not influence the WCA since it remained about 70° up to the longest aging time of 100 min. Longer plasma treatment times in pulses of 10 s each caused slower hydrophobic recovery, but the WCA after 100 min of storage was almost the same as for the sample treated for 30 s. When the samples were stored in a vacuum at room temperature, the hydrophobic recovery was much slower—marginal for the first day, and the highest WCA was observed 5 days after the treatment. The WCA marginally decreased with prolonged storage up to 18 days. The pioneering work by Benisch et al. [28] revealed two facts: one—the aging is faster at higher temperatures, and two—the hydrophobic recovery occurs spontaneously even in vacuum conditions.

Kim et al. [29] are among the first authors who provided systematic results on the aging of plasma-treated LDPE. They used a capacitively coupled RF discharge to sustain oxygen plasma at a pressure of about 100 Pa, and the treatment time was 30 s. The ultimate pressure was about 10^−3^ Pa, and the flow of oxygen through the vacuum chamber was 20 sccm. The samples were films of a thickness of 0.67 mm, and they were placed on the grounded electrode, which was heated to various temperatures during the plasma treatment. The static water contact angle was 85° for untreated LDPE foils. The WCA after the plasma treatment was 52° after treating the sample at 25 °C, and increased monotonously with increasing temperature during the plasma treatment. At a temperature of 100 °C, the WCA was 79° just after plasma treatment, so a marginal increase in the hydrophilicity was achieved by treatment with low-pressure oxygen plasma at elevated temperatures. The poor activation at elevated temperature upon plasma treatment is explained by quick hydrophobic recovery, which was already reported by Benisch et al. [28]. Interestingly enough, the composition of the surface film, as probed by XPS, was almost the same for samples treated at various temperatures between 25 and 100 °C—between 17 and 20 at.% of oxygen. The ATR-FTIR did not reveal significant changes in the spectra of plasma-treated samples, which indicated that the oxygen was concentrated in the surface film of thickness as probed by XPS (several nm). A gradual decrease in the wettability for all treated samples was observed. For the sample treated at 25 °C, the WCA was 48 and 67° after aging at ambient conditions for 1 day and 1 week, respectively. Kim et al. [29] provided a recipe for the suppression of aging using a two-step plasma treatment: In the first step, the LDPE foils are treated in oxygen plasma at a temperature of 100 °C in order to obtain a rather thick layer of oxidized polymers. In the next step, the samples are cooled down to room temperature and treated again with oxygen plasma at room temperature in order to ensure a large concentration of oxygen-containing functional groups on the very surface of the LDPE samples. According to the authors, the two-step process suppressed the migration of oxygen and/or reorientation of surface functional groups, so the aging was much slower because the WCA remained at 56° after a week of aging in ambient conditions. The authors also provided an empirical formula for the behavior of WCA during aging.

**Figure 2:**
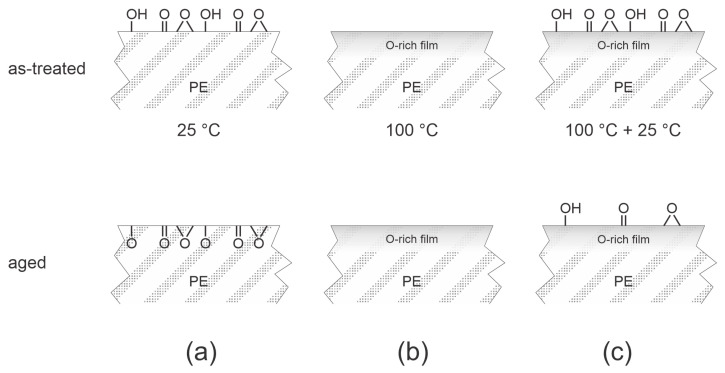
A two-step method for suppression of hydrophobic recovery of oxygen plasma-treated polyethylene (PE) samples. Upper illustrations—as treated, bottom—after accomplishing the hydrophobic recovery. Temperatures during the plasma treatment are indicated just below the upper illustrations, where (**a**) denotes 25 °C, (**b**) 100 °C, and (**c**) 100 + 25 °C.

The two-step procedure recommended by Kim et al. [29] is illustrated in Figure 2. When plasma treatment is performed at room temperature (Figure 2a), the oxygen species from plasma remain on the surface, so functionalization with polar functional groups occurs. The functionalization is not stable because of the re-orientation of polar groups and/or diffusion of oxygen into the sub-surface film, so hydrophobic recovery is pronounced. The instability of surface functional groups is explained by a huge gradient in the oxygen concentration just after the plasma treatment. Namely, the polar groups are formed on the very surface when a polymer is treated at room temperature, and there is no oxygen beneath. Thermodynamics favors the migration of oxygen from large to lower concentrations. The migration will be efficient as long as the gradient is large enough, so a rather fast migration occurs just after the treatment, but the distribution of oxygen concentration stabilizes when a lower gradient is approached. Namely, there is a potential barrier to the migration of oxygen inside the bulk polymer. If plasma treatment is performed at 100 °C (Figure 2b), oxygen will diffuse inside the polymer during the plasma treatment, so O-concentration as deduced by XPS will be large, but the wettability will be poor because of the absence of surface polar groups. The best result (Figure 2c) is obtained through a two-step process. During the first treatment at 100 °C, the O-rich subsurface film is formed. The sample is cooled down to room temperature, and a brief treatment with oxygen plasma will cause the formation of surface groups. The two-step procedure suppresses hydrophobic recovery because the diffusion of oxygen from the surface into the subsurface film is suppressed. This is because the subsurface layer already contains a significant concentration of oxygen, so the gradient in the subsurface film is smaller than in cases when the sample is treated with plasma at room temperature.

Truica-Marasescu et al. [30] treated LDPE with vacuum ultraviolet radiation from a krypton excimer lamp. The source of VUV radiation was low-pressure plasma sustained in pure Kr by an RF discharge at a frequency of 100 MHz. The 35-µm thick polymer foils were mounted into a high vacuum chamber, which was first evacuated below 10^−3^ Pa and then filled with ammonia at the pressure of 40 Pa. The treatment times were up to about one hour, and the authors reported the surface wettability versus the energy dose (a product of the flux of VUV photons, the treatment time, and the photon energy). The absorption of VUV radiation in the gas phase caused weak dissociation of NH_3_ molecules, but major surface modification was caused by the absorption of the VUV radiation in the surface film of the samples. XPS revealed significant functionalization of the surface film as probed by this technique. The concentration of nitrogen and oxygen increased with increasing VUV dose, but it became saturated after receiving a dose of about 5 J/cm^2^ at 24 and 10 at.% of N and O, respectively. The SFE followed the trend of nitrogen and oxygen concentrations. The SFE was about 30 mJ/m^2^ for untreated LDPE, and gradually increased with increasing energy doses to the SFE of about 60 mJ/m^2^ at a photon dose of about 10 J/m^2^. The hydrophobic recovery was studied for three months after the treatment. The surface free energy decreased significantly within the first few days, and then stabilized. The SFE after the three weeks of aging at room temperature were larger for the increased dose of VUV radiation. The storage in the air or under a vacuum caused the same hydrophobic recovery. The WCA was 92° for an untreated LDPE foil and dropped to 12° after treating the foil at a dose of 14 J/cm^2^. The WCA increased to 36, 52, and 68° after storing for 1, 10, and 100 days, respectively.

In another article [31], the same team compared the results obtained by irradiation using VUV photons from the excimer lamp with those obtained by treating the LDPE foils using nitrogen plasma sustained at atmospheric pressure by a DBD discharge operating at a frequency of a few kHz and a peak voltage of 3.7 kV. The treatment time was not reported; instead, the authors reported the energy per unit surface of the polymer samples. The selected energy surface densities were 0.3, 1, and 2 J/cm^2^, the same order as the dose of VUV radiation. The surface free energy of untreated LDPE foils was 27 mJ/m^2^ and increased to 37, 44, and 57 mJ/m^2^ after treating the samples with an energy dose of 0.3, 1, and 2 J/cm^2^, respectively.

**Figure 3:**
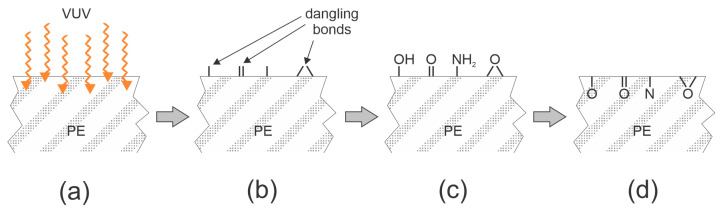
The surface activation and hydrophobic recovery of vacuum ultraviolet (VUV)-treated polyethylene (PE) in moist ammonia atmosphere just after starting the irradiation (**a**). The initial step is formation of surface dangling bonds (**b**), which are rapidly occupied with hydroxyl (–OH), carbonyl (=O), amino (–NH_2_), and epoxy (>O) groups (**c**). Hydrophobic recovery causes re-orientation and/or diffusion of oxygen and nitrogen atoms in the sub-surface film (**d**).

The method disclosed by Truica-Marasescu et al. [30,31] is illustrated in Figure 3. The photon energy from krypton excimers (Kr_2_^*^) peaks at 8.5 eV [32], which is more than any bond in polyethylene. The photons are absorbed in a very thin surface film [7] through bond scission (Figure 3a), so dangling bonds appear on the polymer surface (Figure 3b). The dangling bonds interact with gaseous molecules and form polar functional groups (Figure 3c). The large surface concentration will cause spontaneous reorientation of the functional groups and/or diffusion of O atoms into the sub-surface film, so the hydrophobic recovery is rapid (Figure 3d). Although the authors performed treatment in ammonia, there is always some water vapor in the residual atmosphere of vacuum systems, so the large concentration of O-rich surface functional groups is explained by the interaction of water molecules from the residual atmosphere with the dangling bonds.

Novak et al. [33] treated LDPE of 33% crystallinity with atmospheric pressure plasma sustained in the air by a corona discharge, which operated at a maximum voltage of 9 kV and a frequency of 20 kHz. The additive-free LDPE exhibited an SFE of 48 mJ/m^2^ just after plasma treatment. Storage at ambient conditions caused a gradual decrease in the surface free energy, and the SFE stabilized at about 40 mJ/m^2^ after several months. The same material with a small (less than 0.4 wt.%) addition of various substances, such as phenolic and phosphitic antioxidants, alkyl amine-based antistatic additives, and a slip agent based on the mix of Zn-stearate and Zn-oleate, exhibited a much larger surface free energy after corona plasma treatment at of 55 mJ/m^2^. However, the aging kinetics were similar to that of additive-free LDPE, since the SFE stabilized at 45 mJ/m^2^. The work of adhesion, on the other hand, was much different, since it dropped by a factor of two within a month after the plasma treatment for additive-free LDPE, while it dropped by only about 20% for LDPE with additives. Novak et al. [33] is one of the very few authors who reported the hydrophobic recovery of a plasma-activated polymer determined by two complementary methods: surface energy and adhesion force.

Sanchis et al. [34] treated transparent foils of 50 µm thickness made from LDPE with low-pressure plasma sustained in nitrogen at a pressure of 32 Pa using a capacitively coupled RF discharge. The RF generator operated at 13.56 MHz, and had an output power of 300 W. The vacuum chamber was a cubicle with a dimension of 40 cm. The samples were placed on shelves and were kept floating during the plasma treatment. The aging was performed in a vacuum desiccator at a relative humidity of 10–15% and at room temperature. The authors reported significant weight loss during plasma treatment, at about 10 µg cm^−2^/min. The shortest reported treatment time was 1 min, and caused a drop in the WCA from 90 to 45°. The static water contact angle slowly decreased with increasing treatment time, and reached 38° after treating the polymer sample for 15 min. The hydrophobic recovery did not depend much on the plasma treatment time. The WCA increased monotonously with aging time, and approached about 80° after aging for 170 h. The XPS revealed significant oxidation, but the concentration of nitrogen in the surface film, as probed by XPS, was below 2 at.% for all treatment times. The formation of oxygen-rich functional groups upon plasma treatment is explained by the presence of water vapor in the residual atmosphere and/or inadequate hermetical tightness.

Encinas et al. [35] treated foils made from both low and high-density polyethylene with atmospheric pressure plasma sustained in the air by a plasma torch, which operated at a frequency of 17 kHz and a peak voltage of 20 kV. Plasma-treated foils were stored in dust-free air at a relative humidity of about 50% and a temperature of 25 °C. The untreated LDPE exhibited a surface-free energy of 22 mJ/m^2^, and the plasma treatment caused an increase to 63 mJ/m^2^. No significant deviation from the as-treated samples was observed for the first 20 days of storage, but prolonged aging caused a decrease in the SFE, which assumed about 27 mJ/m^2^ after storing the samples for 270 days. The reported results differ from all previously published papers, since all prior articles reported a rapid decrease in the SFE within the first few days, and a slight (if any) decrease during prolonged aging. The differences are difficult to explain. Similar results were reported in [35] for HDPE. In this case, the SFE of untreated foils was about 27 mJ/m^2^, and increased to 64 mJ/m^2^ after the plasma treatment. No statistically significant variation of the SFE was reported for samples stored for different periods of up to one month. The SFE remained as high as 54 mJ/m^2^ after aging for 270 days. A rather large statistical error (over 10 mJ/m^2^) was reported by Encinas et al. [35].

Jokinen et al. [36] used low-pressure plasma for the treatment of polyethylene (PE300) foils. The plasma reactor was powered by a microwave generator at a frequency of 2.45 GHz at a power of 500 W. The gas flow rate was 800 sccm, but neither the gas pressure nor the pumping speed was provided. The foils were treated with either oxygen or nitrogen plasma. The WCA for untreated PE foils was 95°. Nitrogen plasma treatment for 10 min resulted in a WCA of about 30° and oxygen plasma of about 44°. Some samples were stored in ambient conditions, and others were washed and then stored at ambient conditions. The hydrophobic recovery of all washed samples was almost complete, so the WCA, after prolonged treatment, assumed a value between 80 and 85°. The unwashed samples aged slower, and the final WCA after storing the samples for 120 days was 60–65°. Jokinen et al. [36] therefore showed that the washing causes a significant modification in the surface wettability of plasma-treated PE foils. A feasible explanation for this effect was provided by Kostov et al. [37], who performed the XPS characterization of plasma-treated PE before and after washing in distilled water. They treated PE with an atmospheric pressure plasma jet sustained in argon at atmospheric pressure with a variation of a dielectric barrier discharge (DBD) operating at a frequency of about 30 kHz and a peak-to-peak voltage of 24 kV. The deconvolution of the high-resolution XPS C1s peak of plasma-treated PE samples exhibited about 10% of O−C=O groups, which vanished after rinsing with distilled water.

Hydrophobic recovery of plasma-treated PE immersed into liquids of various molecular dipole moments was also studied by Bormashenko et al. [38]. They exposed extruded low-density polyethylene foils to gaseous plasma sustained at a pressure as low as 0.07 Pa using inductively coupled RF discharge operating at a frequency of 10 MHz and a power of 100 W. The plasma treatment time was 1 min, and resulted in a WCA of about 35°. The samples were immersed in the liquids just after the plasma treatment. They were taken from the liquids and dried in vacuum conditions before measuring the water contact angles. The reported kinetics of the hydrophobic recovery depended on the polarity of the chosen liquid. As a rule of thumb, the hydrophobic recovery was rendered when using polar liquids, including water. The WCA of samples aged in liquids for one day at 8 °C were 47 and 105° for acetone and carbon disulfide, respectively. The reported molecular dipole moments were 2.88 and 0.0 Debyes for (CH_3_)_2_CO and CS_2_, respectively. Storage in the same liquid for one day resulted in a WCA of 55 and 95°, respectively. Some samples were also stored in the air at ambient conditions or in a vacuum chamber (without immersing into the liquids), and the hydrophobic recovery was more gradual. The WCA for samples stored in the air was 62 and 77° after 1 and 7 days, respectively, while for those stored in the vacuum, the WCA was 55 and 57°, respectively. The results reported by Bormashenko et al. [38] differ from those reported by Jokinen et al. [36], because Jokinen reported a quick hydrophobic recovery of plasma-treated samples immersed into a polar liquid (water). The paradox has yet to be explained.

Corona discharge was used for plasma treatment of LDPE by Lindner et al. [39]. They treated LDPE foils in air plasma at power doses between about 1 and 5 J/cm^2^. The surface free energy of untreated samples was about 22 mJ/cm^2^ and increased to 36 mJ/cm^2^ after receiving an energy dose of 1 J/cm^2^. The SFE increased slowly with increasing energy doses, and reached 45 mJ/cm^2^ at 5 J/cm^2^. The plasma-treated samples were stored at ambient conditions, and the evolution of the hydrophobic recovery was measured occasionally. The loss of wettability was found to be linear with storage time, and depended on the energy dose received upon plasma treatment. It was between 0.03 and 0.07 mJ/cm^2^ per day. The adhesion force was measured for plasma-treated samples (without aging), and was found to increase steeply with increasing power doses: it was only 2.5 N for samples treated at a power dose of 1 J/cm^2^, and about 7 N for 5 J/cm^2^.

Matoušek et al. [40] treated PE foils in air plasma sustained at atmospheric pressure by a DBD discharge powered using an AC source operating at a frequency of 3 kHz, a peak voltage of 20 kV, and a power of 120 W. The static water contact angle of untreated samples was about 95°. A treatment time of 1 s caused a drop in the WCA to 66°, and a treatment time of 2 s caused a drop to about 60°. No decrease in the WCA was observed for longer treatment times. The samples were aged at ambient conditions, and gradual hydrophobic recovery was observed within the first few days. The prolonged aging did not influence the WCA, since it remained close to 90° for all plasma treatment times from 10 to 60 days. The authors also measured the composition of the surface film as probed by XPS, and reported large scatterings in the oxygen concentration with storage time. The hydrophobic recovery influenced the adhesion of paint on the PE samples. The adhesion force for as-treated samples was almost three times that for untreated samples, but the forces were practically the same after storing samples for a month or longer.

The results of all authors who studied the hydrophobic recovery of plasma-treated polyethylene are summarized in Table 1 and Table 2. Table 1 summarizes the literature that reported the water contact angle, and Table 2 summarizes results where the surface free energy was reported.

The values of WCA and SFE (Table 1 and Table 2, respectively) for untreated polyethylene reported by different authors vary significantly. For example, the lowest WCA for low-density polyethylene was 85°, reported by Kim et al. [29], and the largest was 97°, reported by Benisch et al. [28]. The WCA depends on the synthesis procedure, including the purity, any surface impurities, the roughness, and the accuracy of the drop-shape analyzer. The same applies for the surface free energy. The lowest SFE for untreated samples of 22 mJ/m was reported by Encinas et al. [35] and Lindner et al. [39], while Truica-Marasescu et al. [30] found the SFE for untreated PE samples as large as 30 mJ/m. The scattering of the results reported by various authors should be taken into account at any attempt to interpret the aging phenomenon of plasma-treated polymer samples.

## 3. Correlations

As described above, different authors employed different experimental systems, so it is difficult to compare the results. The surface finish definitely depends on the type and fluxes of reactive plasma species and the photon energies, as well as the flux of photons with energy higher than the binding energies in the polymer. Unfortunately, only a few authors reported these parameters, so it is not possible to draw correlations between the fluxes (or fluences) of plasma species and the evolution of surface wettability. Still, the correlations between the processing parameters and the surface wettability are useful because they enable drawing general trends.

**Figure 4:**
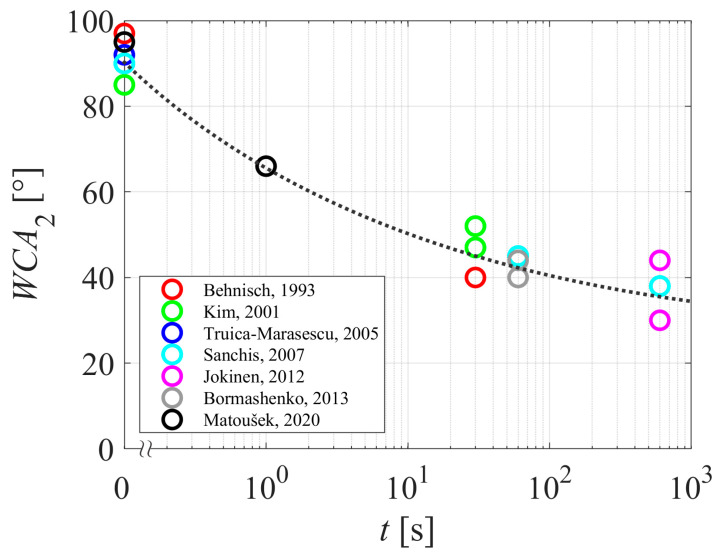
The WCA reported in different articles of PE samples after plasma treatment versus the plasma treatment time. The dotted curve is for eye guidance. References: [28,29,30,34,36,38,40].

**Figure 5:**
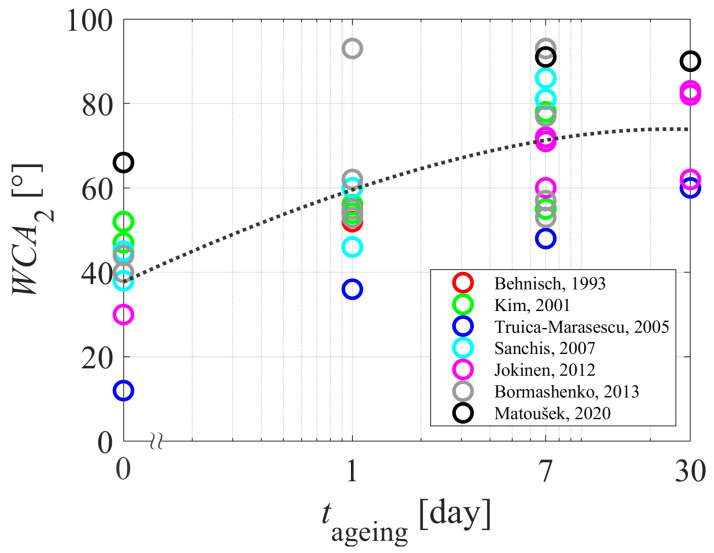
The WCA versus the aging time. The dotted curve is for eye guidance. References: [28,29,30,34,36,38,40].

**Figure 6:**
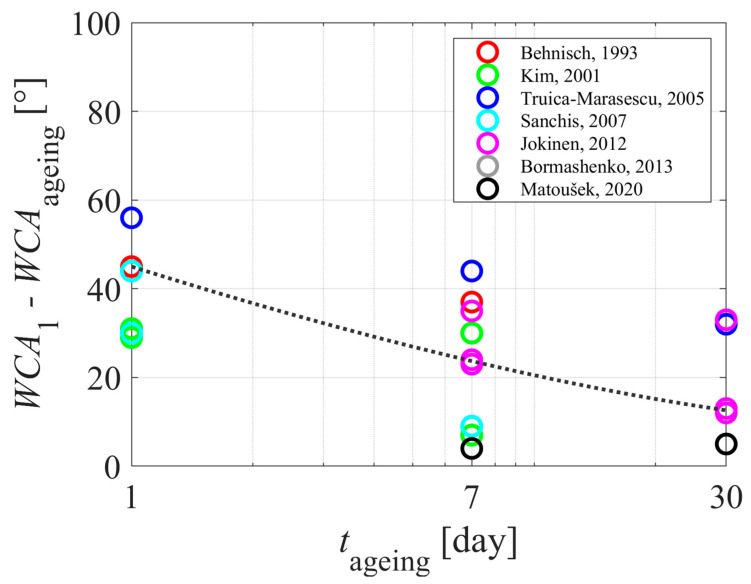
The difference between the WCA of untreated samples and WCA of plasma-treated samples versus the aging time. The dotted curve is for eye guidance. References: [28,29,30,34,36,38,40].

Table 1 and Table 2 summarize the reported results on the aging of plasma-hydrophilized polyethylene samples. The evolution of the WCA measured just after treating the samples with gaseous plasma is plotted against the treatment time in Figure 4. The WCA versus the aging time of the polyethylene samples is plotted in Figure 5. Different authors reported different WCA for untreated samples, so it pays to normalize the WCA of aged samples with the value reported for untreated samples. The difference between the WCA of untreated samples and WCA after aging is plotted in Figure 6.

Figure 4 represents the water contact angle of the polyethylene samples treated in gaseous plasma for different periods. Despite the fact that the authors used a variety of experimental setups, the correlation is straightforward taking into account the scattering of the reported WCA values: the WCA decreases monotonously with increasing treatment time. Taking into account the log scale on the *x*-axis and the linear scale on the *y*-axis, the behavior is far from linear—the hydrophilization occurs already at short treatment times, and the decrease in the WCA is slower at longer treatment times. The effect is explained using the kinetics of plasma treatment (Figure 1 and Figure 2). The surface functionalization occurs quickly, but the diffusion of oxygen into the subsurface film and/or reorientation of the surface functional groups occurs due to the large gradient in the oxygen concentration in the surface film. Prolonged treatment allows diffusion already during the plasma treatment, so the WCA measured just after the treatment decreases with increasing treatment time. The effect was best illustrated by Kim et al. [29], who clearly showed the influence of oxygen diffusion on the wettability of the PE samples.

The aging of plasma-treated polymer samples is revealed in Figure 5. Again, the results are scattered, but the trend is obvious: the WCA increases with increasing storage time. The WCA, although perhaps one of the simplest experimental techniques, is therefore useful for studying the aging of plasma-treated PE samples due to the hydrophobic recovery. Huge scatterings of the results reported by various authors indicate the complexity of the aging phenomenon. As illustrated in Figure 2 and Figure 3, the hydrophobic recovery definitely depends on the thickness of the oxygen-rich surface film formed upon plasma treatment. The thickness, however, has not been reported in the reviewed literature. Some authors probed the surface using XPS, and did not find a straightforward correlation between the oxygen content as determined by XPS and the wettability as determined by WCA. This observation may lead to the conclusion that the thickness of the oxygen-rich film should be larger than the escape depth of photoelectrons from C1s level (several nm). The measured points in Figure 6 are somehow less scattered than in Figure 5, which is explained by the differences in the initial WCA (before the plasma treatment) reported by different authors.

**Figure 7:**
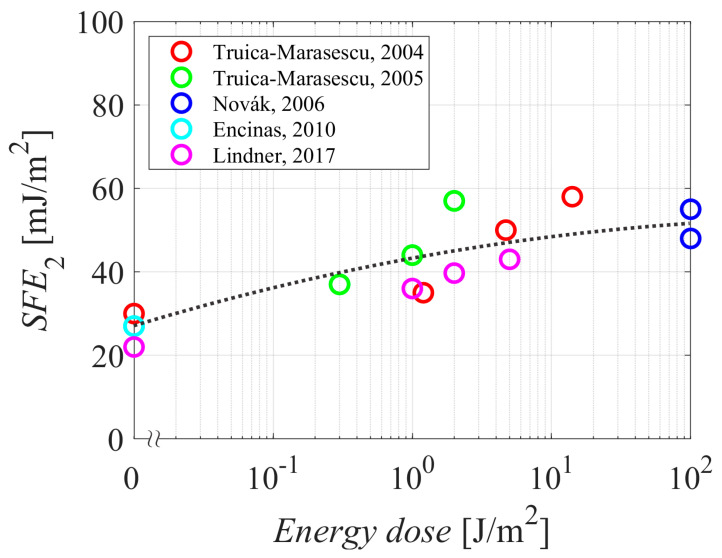
The SFE of PE samples after plasma treatment versus the energy dose. The dotted curve is for eye guidance. References: [30,31,33,35,39].

Several authors reported the SFE instead of WCA. These authors did not mention the plasma treatment time but rather the energy dose (plasma energy per unit surface area). Figure 7 represents the SFE measured just after treating the samples with gaseous plasma versus the energy dose. As explained above, the energy dose is the discharge power divided by the width of the glowing plasma and the speed of moving the polymer sample through the plasma. The authors who reported the SFE performed the treatment either in nitrogen or air, and all used atmospheric pressure discharges. The majority of the discharge power in atmospheric-pressure discharges is spent on gas heating, which is a consequence of the three-body collisions between the atoms and/or excited molecules, so it is difficult to evaluate the power spent on the exothermic surface reactions such as functionalization of PE samples and etching. Still, the trend is obvious—the SFE increases with the increasing energy dose. The behavior is explained as above for the case of the treatment time—the thickness of the oxygen-rich surface film increases with increasing energy doses. Figure 7 also reveals that a very large energy dose does not cause a further increase in the SFE, so it seems to be saturated at a value of roughly 50–60 mJ/m^2^.

**Figure 8:**
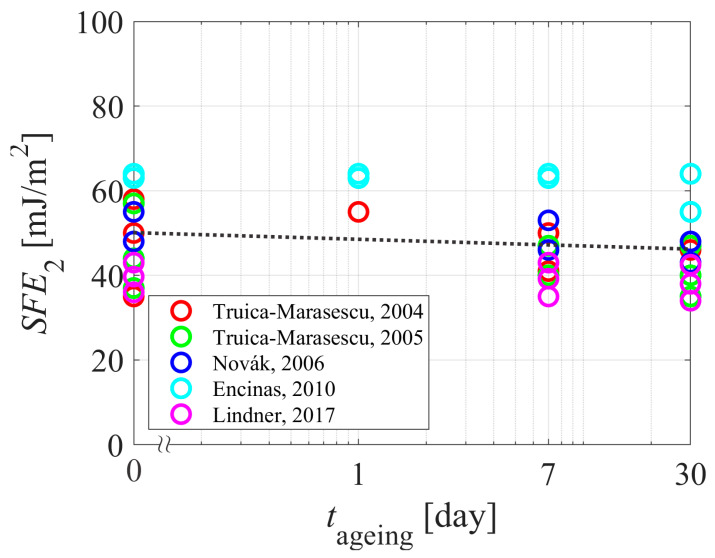
The SFE versus the aging time. The dotted curve is for eye guidance. References: [30,31,33,35,39].

**Figure 9:**
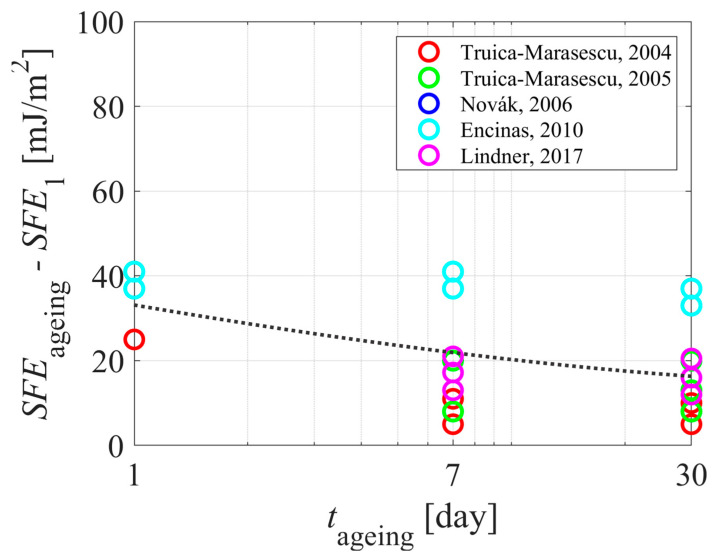
The difference between the SFE of plasma-treated samples and the SFE of untreated samples versus the aging time. The dotted curve is for eye guidance. References: [30,31,33,35,39].

Figure 8 shows the decrease of the SFE upon aging, and Figure 9 shows the values normalized to the initial SFE; i.e., the SFE of untreated polyethylene samples. Surprisingly enough, the decrease of the SFE during aging (Figure 8) is marginal, indicating that measuring the surface free energy is not the appropriate method for monitoring the aging of the PE polymer due to the hydrophobic recovery. The paradox may be explained by different initial values of the surface free energy (before the plasma treatment). The normalized aging values—i.e., the difference between the SFE of aged plasma-treated samples and the SFE of untreated samples versus the aging time (Figure 9)—better represents the aging phenomenon because the generalized curve shows a monotonous decrease of the surface free energy with increasing aging time. Still, the general curve shown in Figure 6 is more persuading than the curve in Figure 9. A possible explanation is that the surface free energy is a sum of the dispersive and the polar components. Both may change as a result of the plasma treatment, which brings another uncertainty in the reported values. Any discussion about the reasons for changing the dispersive component of the surface free energy upon plasma treatment is beyond the scope of this paper, but the illustration in Figure 1 shows that not only the surface polarity, but the other properties of the polymer may also change during plasma treatment due to the irreversible modification of the surface film by VUV photons. In any case, the comparisons between Figure 5 and Figure 8 or between Figure 6 and Figure 9 show that the WCA method provides a more distinct method for evaluation of the aging phenomena of plasma-hydrophilized PE than SFE.

## 4. Conclusions

The aging of plasma-induced hydrophilization of polyethylene represents a serious obstacle for broader application, so there is a need to invent methods for evaluating the aging phenomenon, as well as suppressing the aging by storing the plasma-treated samples at appropriate conditions. A trivial solution is keeping the plasma-treated samples at a very low temperature. Namely, the hydrophobic recovery is usually attributed to the mobility of functional groups and diffusion, and both increase with increasing temperature. However, it is known that storing at low temperature makes common plastics become brittle.

An alternative is formation of a thin surface film rich in oxygen upon plasma treatment. The authors have not examined this procedure systematically, but the correlations clearly indicate that a thicker oxide film should suppress hydrophobic recovery. This is explained by suppressed diffusion of oxygen from the surface into the sub-surface film in cases where the sub-surface film contains a significant amount of oxygen, so the oxygen gradient is not as large as for the cases where only the very thin oxygen-containing film is formed. The thicker oxide film is achieved either by treating polymers in plasma at an elevated temperature or by prolonged treatment. Unfortunately, only one author reported the temperature of the PE samples during the plasma treatment. The surface reactions are exothermic, so the surface temperature definitely increases with increasing treatment time and/or the energy dose. The power dissipated on the polymer surface upon exposure to oxygen-containing plasma increases with increasing doses of reactive species, so the fluxes will be needed in order to estimate the surface temperature. Unfortunately, the authors have not reported the fluxes. As mentioned earlier, and according to Figure 1, the major reactants causing exothermic surface reactions are positively charged ions, neutral oxygen atoms in the ground state, and energetic photons. The ions will neutralize on the polymer surface, and will dissipate their kinetic energy gained when passing the sheath, so the dissipated energy is the sum of their kinetic and ionization energy. The neutral atoms will associate on the polymer surface by heterogeneous surface recombination, so the dissociation energy will be spent on the surface heating. This effect could be evaluated if the probability for surface recombination is known.

Yet another alternative is the fixation of surface functional groups by storing the polymer in a liquid. There is a huge discrepancy between authors who probed the hydrophobic recovery upon storage in liquids. For example, in their classical work, Jokinen et al. [36] reported almost immediate and complete hydrophobic recovery of PE foils after rinsing with distilled water. In contrast, Bormashenko et al. [38] reported stable hydrophilicity upon storage for a prolonged time in polar liquids, including water. The influence of the storage liquid on the hydrophobic recovery of plasma-hydrophilized polyethylene thus remains a scientific challenge.

A promising technique to reverse hydrophobic recovery could be using surface dynamics to cycle surface properties by alternating storage in polar/non-polar media [41].

## Figures and Tables

**Table 1:** The summary of experimental conditions and reported hydrophobic recovery of plasma-treated polyethylene samples and the increase of the WCA after aging for different times.

Author	PE Type	Discharge	*p* [Pa], Gas	*p* [W]/Dose [J/m^2^]	WCA ^1^	WCA ^2^	10 Min	1 h	1 Day	1 Week	1 Month	Ref.	Comment	*t*_treatment_
Behnisch	PE	RF-CCP AG	50, O_2_	N/A	97	40	70	70	N/A	N/A	N/A	[28]	Stored in vacuum at 50 C	30 s
Behnisch	PE	RF-CCP	50, O_2_	N/A	97	40	N/A	N/A	52	60	N/A	[28]	Stored in vacuum at RT	30 s
Kim	LDPE	RF-CCP	100, O_2_	25	85	52	N/A	N/A	56	78	N/A	[29]	One-step	30 s
Kim	LDPE	RF-CCP	100, O_2_	25	85	47	N/A	N/A	54	55	N/A	[29]	Two-step	30 s
Truica-Marasescu	LDPE	VUV, Kr	40, NH_3_	D14.1	92	12	N/A	N/A	36	48	60	[30]	/	N/A
Sanchis	LDPE	RF-CCP	32, N_2_	300	90	45	N/A	N/A	46	86	N/A	[34]	/	1 min
Sanchis	LDPE	RF-CCP	32, N_2_	300	90	38	N/A	N/A	60	81	N/A	[34]	/	10 min
Jokinen	PE	MW	N/A, O_2_	500	95	44	N/A	N/A	N/A	60	62	[36]	Stored in air	10 min
Jokinen	PE	MW	N/A, O_2_	500	95	44	N/A	N/A	N/A	71	83	[36]	Stored in water	10 min
Jokinen	PE	MW	N/A, N_2_	500	95	30	N/A	N/A	N/A	60	62	[36]	Stored in air	10 min
Jokinen	PE	MW	N/A, N_2_	500	95	30	N/A	N/A	N/A	72	82	[36]	Stored in water	10 min
Bormashenko	PE	RF-ICP	0.07, N/A	100	N/A	44	N/A	N/A	62	77	N/A	[38]	Stored in water	1 min
Bormashenko	PE	RF-ICP	0.07, N/A	100	N/A	44	N/A	N/A	55	57	N/A	[38]	Stored in vacuum	1 min
Bormashenko	PE	RF-ICP	0.07, N/A	100	N/A	44	N/A	N/A	53	53	N/A	[38]	Stored in acetone	1 min
Bormashenko	PE	RF-ICP	0.07, N/A	100	N/A	40	N/A	N/A	93	93	N/A	[38]	Stored in CS_2_	1 min
Matoušek	PE	DBD	105, air	120	95	66	N/A	N/A	N/A	91	90	[40]	Stored in air	1 s
Average values	/	/	/	/	92.58	41.25	/	/	56.7	69.47	73.17	/	/	/

WCA ^1^ is the initial water contact angle, WCA ^2^ is the water contact angle just after plasma treatment, RF-CCP is the capacitively-coupled radiofrequency plasma, AG is the plasma afterglow, VUV is the vacuum ultraviolet radiation, MW is the microwave plasma, RF-ICP is the inductively-coupled radiofrequency plasma, DBD is the dielectric barrier discharge plasma, RT is room temperature, N/A is not available.

**Table 2:** The summary of experimental conditions and reported hydrophobic recovery of plasma-treated polyethylene samples and the decrease of the SFE after aging for different times. All authors reported aging in the air at ambient conditions. The plasma treatment times were not reported. Instead, the energy dose was reported.

Author	PE Type	Discharge	*p* [Pa]	Energy Dose [J/m^2^]	SFE ^1^ [mJ/m^2^]	SFE ^2^ [mJ/m^2^]	1 Day	1 Week	1 Month	Ref.
Truica-Marasescu	LDPE	VUV, Kr	40	1.2	30	35	N/A	35	35	[30]
Truica-Marasescu	LDPE	VUV, Kr	40	4.7	30	50	N/A	41	40	[30]
Truica-Marasescu	LDPE	VUV, Kr	40	14.1	30	58	55	50	46	[30]
Truica-Marasescu	LDPE	DBD, N_2_	10^5^	0.3	27	37	N/A	35	35	[31]
Truica-Marasescu	LDPE	DBD, N_2_	10^5^	1	27	44	N/A	40	40	[31]
Truica-Marasescu	LDPE	DBD, N_2_	10^5^	2	27	57	N/A	47	47	[31]
Novak	LDPE pure	Corona, air	10^5^	100	N/A	48	N/A	46	43	[33]
Novak	LDPE w. additive	Corona, air	10^5^	100	N/A	55	N/A	53	48	[33]
Encinas	LDPE	RF, air	10^5^	N/A	22	63	63	63	55	[35]
Encinas	HDPE	RF, air	10^5^	N/A	27	64	64	64	64	[35]
Lindner	LDPE	Corona, air	10^5^	1	22	36	N/A	35	34	[39]
Lindner	LDPE	Corona, air	10^5^	2	22	39.7	N/A	39.2	38	[39]
Lindner	LDPE	Corona, air	10^5^	5	22	43	N/A	43	42.5	[39]
Average values	/	/	/	/	26	48.44	60.67	45.48	43.65	/

SFE ^1^ is the initial surface free energy, SFE ^2^ is the surface free energy just after plasma treatment, VUV is the vacuum ultraviolet radiation, DBD is the dielectric barrier discharge plasma, RF is radiofrequency, N/A is not available.

## Data Availability

This is a review article; therefore, no data were created.
